# Efficient Degradation of Acesulfame by Ozone/Peroxymonosulfate Advanced Oxidation Process

**DOI:** 10.3390/molecules24162874

**Published:** 2019-08-08

**Authors:** Yu Shao, Zhicheng Pang, Lili Wang, Xiaowei Liu

**Affiliations:** 1 Institute of Municipal Engineering, College of Civil Engineering and Architecture, Zhejiang University, Hangzhou 310058, China; 2 Institute of Water Resources & Ocean Engineering, Ocean College, Zhejiang University, Hangzhou 310058, China; 3 Environmental Engineering, Jiyang College of Zhejiang A & F University, Zhuji 311800, China

**Keywords:** ozone, peroxymonosulfate, acesulfame, advanced oxidation, sulfate radical

## Abstract

Artificial sweeteners (ASWs), a class of emerging contaminants with good water solubility, have attracted much attention recently because of their wide use and negative impact on the aquatic environment and drinking water. Efficient technologies for removing ASWs are in urgent need. This study investigated degradation of typical ASW acesulfame by ozone-activated peroxymonosulfate process (O_3_/PMS) in prepared and real waters. O_3_/PMS can degrade >90% acesulfame in prepared water within 15 min at a low dosage of O_3_ (60 ± 5 µg∙min^−1^) and PMS (0.4 mM). Ozone, hydroxyl radical (HO•), and sulfate radical (SO_4_•^−^) were identified as contributors for ACE degradation and their contribution proportion was 27.1%, 25.4%, and 47.5% respectively. O_3_/PMS showed the best degradation performance at neutral pH and were sensitive to constituents such as chloride and natural organic matters. The qualitative analysis of degradation products confirmed the involvement of hydroxyl radical and sulfate radical and figured out that the active sites of ACE were the C=C bond, ether bond, and C-N bond. The electrical energy per order ACE degradation were calculated to be 4.6 kWh/m^3^. Our findings indicate that O_3_ is an efficient PMS activator and O_3_/PMS is promising due to its characteristic of tunable O_3_^−^HO• SO_4_•^−^ ternary oxidant involving.

## 1. Introduction

As a class of emerging pollutant, artificial sweeteners (ASs), have recently received increasing attention [1,2,3]. ASs are synthetic or semi-synthetic organic compounds that replace sucrose and are widely used in food, beverage, pharmaceutical, and personal care products [4]. Most artificial sweeteners are hardly converted by the human body (called as non-caloric sugars) and are generally highly water-soluble. Thus, the aqueous environment is their main destination. There are more than 20 kinds of ASs currently used, and there are five kinds of sweeteners that are often considered in the water environment, namely saccharin (SAC), cyclamate (CYC), aspartame (ASP), acesulfame (ACE), and sucralose (SUC) [5]. The high water solubility, large amount of use, and anti-biodegradation property (SAC, CYC, ACE, and SUC biodegradation cycle > 15d, [6]) of ACSs make them frequently detected in surface water [2], groundwater [7], drinking water [6,8], and sewage treatment plant effluent [2]. Concentration of ASs in drinking water has reported to be tens of ng·L^−1^ to several hundred μg·L^−1^, which is much higher than that of other emerging pollutants such as drugs and personal care products and endocrine disruptors. The toxicology of ACSs is not clear yet, but its negative effects on the human health have been reported [9].

Given the limited capacity of conventional water treatment (coagulation–sedimentation–filtration–chlorination) to remove ACSs [6], researchers evaluated their enhanced removal by advanced technologies including ozone/permanganate/ferrate oxidation [10,11,12], activated carbon/metal organic framework materials/magnetic ion exchange resin adsorption [1,13], UV photolysis [14], advanced oxidation [15,16,17], and membrane filtration [18]. Among these technologies, advanced oxidation processes (AOPs) and reverse osmosis (RO) were proved to work best (~10^−2^ min^−1^ for AOPs and rejection rate > 90% for RO). In terms of operating and maintenance costs, degradation of ACSs by AOPs seems to be more attractive. 

In recent years, sulfate radical-based advanced oxidation processes (${\mathrm{SO}}_{4}^{•-}$-AOPs) have received much attention for their efficient destruction of organic contaminants [19,20]. Sulfate radical is a strong oxidant (2.5−3.1 V) and reacts with many organic pollutants at nearly diffusion-controlled rates, which are comparable to hydroxyl radical (HO•). The reactions of ${\mathrm{SO}}_{4}^{•-}$ with organic compounds primarily follow a one-electron transfer mechanism [21], which facilitates the decarboxylation reactions and thus leads to a more efficient mineralization performance than HO•. Additionally, ${\mathrm{SO}}_{4}^{•-}$, compared to HO•, is less influenced by competing constituents in water background matrix, such as bicarbonate and natural organic matter in real water [21], implying that ${\mathrm{SO}}_{4}^{•-}$ is more favorable to destruct high reactive organic contaminants.

As a common precursor of ${\mathrm{SO}}_{4}^{•-}$, peroxymonosulfate (PMS) is often used to produce ${\mathrm{SO}}_{4}^{•-}$ in presence of activators [19]. PMS can be activated by UV, transition metals, heat, base, zero-valent metal, activated carbon, quinones, ultrasonication, gamma radiation, glucose, metal oxides, and electron donated electrochemical system [22]. A recent study found that ozone (O_3_) can also activate PMS to produce ${\mathrm{SO}}_{4}^{•-}$ and a possible mechanism was proposed (Equations (1)–(6), [23,24]). The mechanism is that ozone reacts with PMS (${\mathrm{HSO}}_{5}^{-}$/${\mathrm{SO}}_{5}^{2-}$) to generate ${\mathrm{SO}}_{8}^{2-}$, which quickly decompose into precursors of ${\mathrm{SO}}_{4}^{•-}$ and HO• (${\mathrm{SO}}_{5}^{•-}$ and $O_{3}^{•-}$). However, the fundamental aspects of this ${\mathrm{SO}}_{4}^{•-}$-AOP (e.g., influence of operational and water quality parameters and contribution quantitative analysis of radicals) have not been clarified yet.

(1) $${\mathrm{SO}}_{5}^{2-}+O_{3}\to {\mathrm{SO}}_{8}^{2-}$$

(2) $${\mathrm{SO}}_{8}^{2-}\to {\mathrm{SO}}_{5}^{•-}+O_{3}^{•-}$$

(3) $${\mathrm{SO}}_{5}^{•-}+O_{3}\to {\mathrm{SO}}_{4}^{•-}+2O_{2}$$

(4) $${\mathrm{SO}}_{5}^{•-}+{\mathrm{SO}}_{5}^{•-}\to 2{\mathrm{SO}}_{4}^{•-}+O_{2}$$

(5) $$O_{3}^{•-}\to O^{•-}+O_{2}$$

(6) $$O^{•-}+H_{2}O\to {\mathrm{HO}}^{•}+{\mathrm{OH}}^{-}$$

To fill the abovementioned gaps, the ACE degradation by O_3_/PMS was particularly focused in this study. The contributions of the reactive oxidative species were distinguished. Moreover, the mineralization rate and degradation products were detected. Specifically, the degradation behaviors in real waters (four effluent of filter tank of waterworks) were tentatively studied for the first time. Then, the influence of operational parameters (dosage of O_3_ and PMS) and common water quality parameters (solution pH, bicarbonate, chloride, and natural organic materials (NOM)) on the degradation processes was systematically investigated. Finally, the economic cost was evaluated.

## 2. Results and Discussion

### 2.1. Degradation Effeciency of ACE by O_3_/PMS

**Figure 1:**
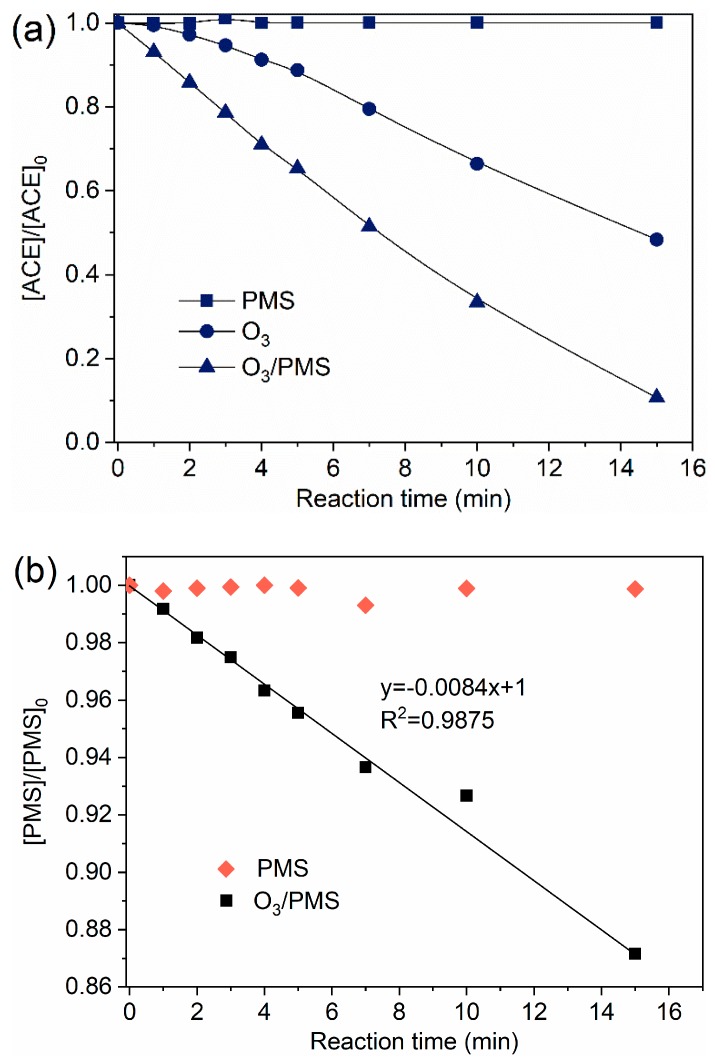
Degradation of ACE by different processes: (**a**) Evolution of ACE normalized concentration ([ACE]/[ACE]_0_); (**b**) Evolution of PMS normalized concentration ([PMS]/[PMS]_0_). Conditions: [ACE]_0_ = 8.0 mg·L^−1^; pH = 7.4; 15 ± 1 °C; O_3_ solution dosing rate 1.25 ± 0.1 µM∙min^−1^ (60 ± 5 µg∙min^−1^); [PMS]_0_ = 0.4 mM).

ACE degradation by O_3_, PMS, and O_3_/PMS were compared. The results are presented in Figure 1a. PMS oxidation alone nearly did not degrade ACE for 15 min reaction time, while O_3_ oxidation showed a 52.7% degradation rate of ACE at the same reaction time. The fastest ACE degradation (90.4%) was observed in O_3_/PMS system. O_3_ oxidation usually includes direct oxidation (pollutants react with O_3_ molecular directly) and indirect oxidation (pollutants react with radicals generated from O_3_ decomposition). Given the high oxidation potential of O_3_ (2.07 V), direct oxidation of O_3_ was believed to play an important role. As O_3_ can activate PMS to produce ${\mathrm{SO}}_{4}^{•-}$ and HO• (Equations (1)–(6)), the excellent degradation performance of O3/PMS process may be also contributed by these two oxidative radicals. The activation effect of O_3_ on PMS was confirmed by the accelerated PMS decomposition rate in the presence of O_3_ (Figure 1b). 

**Figure 2:**
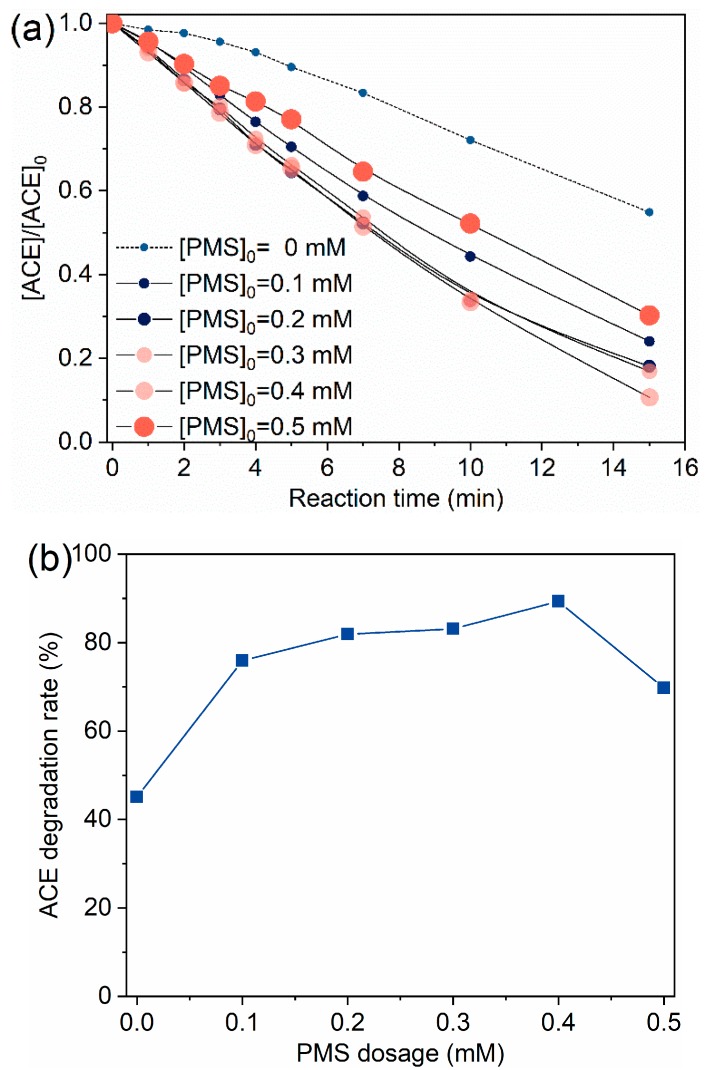
Degradation of ACE by O_3_/PMS at different PMS dosage: (**a**) Evolution of ACE concentration; (**b**) ACE degradation rate vs. PMS dosage. Conditions: [ACE]_0_ = 8.0 mg·L^−1^; pH = 7.4; 15 ± 1 °C; O_3_ solution dosing rate 1.25 ± 0.1 µM∙min^−1^ (60 ± 5 µg∙min^−1^).

Figure 2 displays the effects of PMS dosage on ACE degradation by O_3_/PMS. ACE degradation was promoted when the PMS dosage increased from 0.1 mM to 0.4 mM, and the further increase of PMS dosage resulted in a decreased degradation of ACE (Figure 2a). That is, 0.4 mM PMS combining with O_3_ dosing 60 ± 5 µg∙min^−1^ showed the best degradation of ACE (Figure 2b). Because ozone was dosed in a continuous way and PMS was added in one time, the scavenging effect of HO• by PMS (${\mathrm{HSO}}_{5}^{-}$) is expected to be more and more significant with the dosage increase of PMS (Equations (7)–(10), [25,26,27,28]) and consequently leads to a deteriorating degradation performance. Therefore, a PMS dosage of 0.4 mM was used in the following experiments.

(7) $${\mathrm{HSO}}_{5}^{-}\to {\mathrm{SO}}_{5}^{2-}+H^{+}\;pKa=9.4$$

(8) $$\mathrm{HO}+{\mathrm{HSO}}_{5}^{-}\to {\mathrm{SO}}_{5}^{•-}+H_{2}O\;1.7\times 10^{7}{\;M}^{-1}\cdot s^{-1}$$

(9) $${\mathrm{SO}}_{5}^{2-}+\mathrm{HO}\to {\mathrm{SO}}_{5}^{•-}+{\mathrm{OH}}^{-}2.1\times 10^{9}{\;M}^{-1}\cdot s^{-1}$$

(10) $${\mathrm{SO}}_{4}^{•-}+{\mathrm{HSO}}_{5}^{-}\to {\mathrm{SO}}_{5}^{•-}+{\mathrm{HSO}}_{4}^{-}\;<10^{5}{\;M}^{-1}\cdot s^{-1}$$

(11) $$\mathrm{HO}+O_{3}\to {\mathrm{HO}}_{2}^{•}+O_{2}\;1.0\times 10^{8}{\;M}^{-1}\cdot s^{-1}$$

(12) $${\mathrm{SO}}_{4}^{•-}+O_{3}\to {\mathrm{SO}}_{5}^{•-}+O_{2}\;\mathrm{Slow}$$

### 2.2. Degradation Mechanism

#### 2.2.1. Contributions of Different Reactive Species

**Figure 3:**
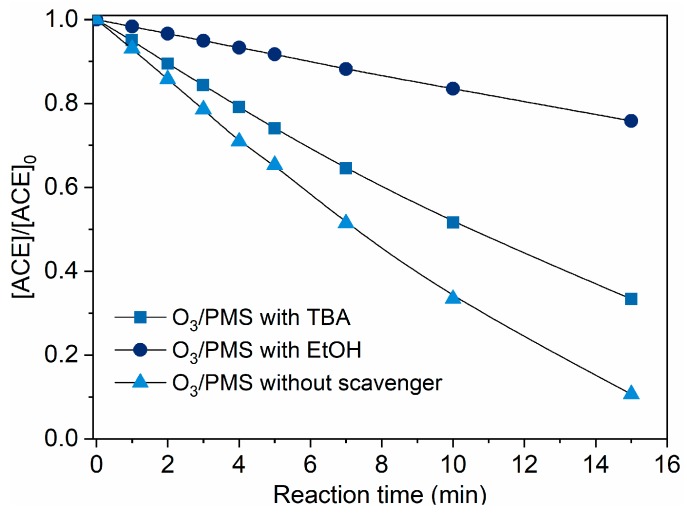
Degradation of ACE by O_3_/PMS in the presence of different scavengers. Conditions: [ACE]_0_ = 8.0 mg·L^−1^; pH = 7.4; 15 ± 1 °C; O_3_ dosing rate 1.25 ± 0.1 µM∙min^−1^ (60 ± 5 µg∙min^−1^); [PMS]_0_ = 0.4 mM; [TBA]_0_ = 0.4 mM; [EtOH]_0_ = 0.4 mM).

**Figure 4:**
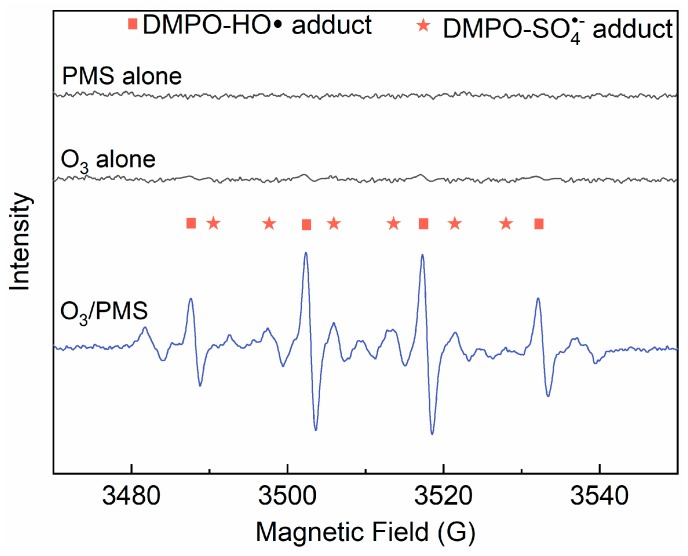
Derivative electron paramagnetic resonance (EPR) spectra of samples collected from PMS alone, O_3_ alone, and O_3_/PMS systems. Conditions: [PMS]_0_ = 0.4 mM; [DMPO]_0_ = 1.0 g·L^−1^; [O_3_]_0_ = 41.7 µM (2 mg·L^−1^); pH = 7.4; 15 ± 1 °C).

Based on the above discussion, we can preliminarily assume that ACE degradation mainly contributed by direct O_3_ oxidation and ${\mathrm{SO}}_{4}^{•-}$/ HO• attack. To clarify this issue, TBA (HO• scavenger, [19]) and EtOH (scavenger of both HO• and ${\mathrm{SO}}_{4}^{•-}$, [19]) were introduced into the O_3_/PMS system. As shown in Figure 3, the addition of TBA and MeOH made the ACE degradation decrease by 22.7% and 65.1%, respectively. Thus, the contributions of direct O_3_ oxidation, HO• oxidation, and ${\mathrm{SO}}_{4}^{•-}$ oxidation are 24.2%, 22.7%, and 42.4%, which corresponds a ratio of 27.1: 25.4: 47.5. A direct support from EPR testing results confirmed the formation of HO• and ${\mathrm{SO}}_{4}^{•-}$ (Figure 4). 

#### 2.2.2. Degradation Products

**Figure 5:**
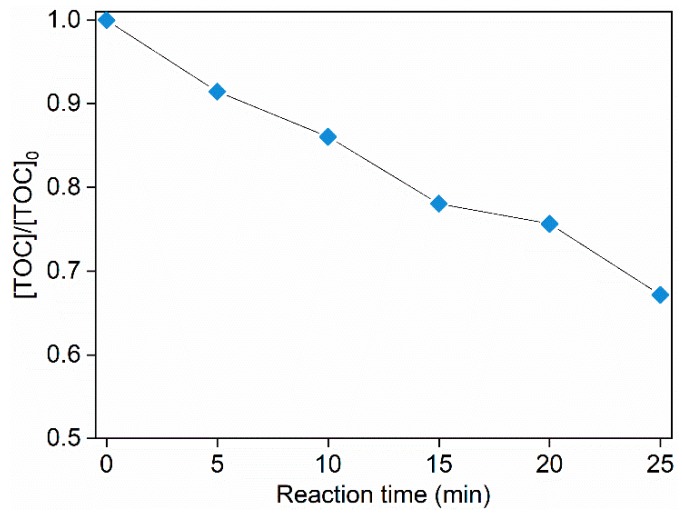
TOC evolution during degradation of ACE by O_3_/PMS. Conditions: [ACE]_0_ = 8.0 mg∙L^−^^1^; pH = 7.4; 15 ± 1 °C; O_3_ dosage 1.25 ± 0.1 µM∙min^−1^ (60 ± 5 µg∙min^−1^); [PMS]_0_ = 0.4 mM.

Considering that the degradation products of an oxidation system are usually highly associated with the oxidative species, degradation products of ACE by O_3_/PMS were determined through HPLC-MS to testify the participation of HO• and ${\mathrm{SO}}_{4}^{•-}$. The 32.8% total organic carbon (TOC) removal rate (Figure 5) indicates that many transformation intermediates are generated. 

**Figure 6:**
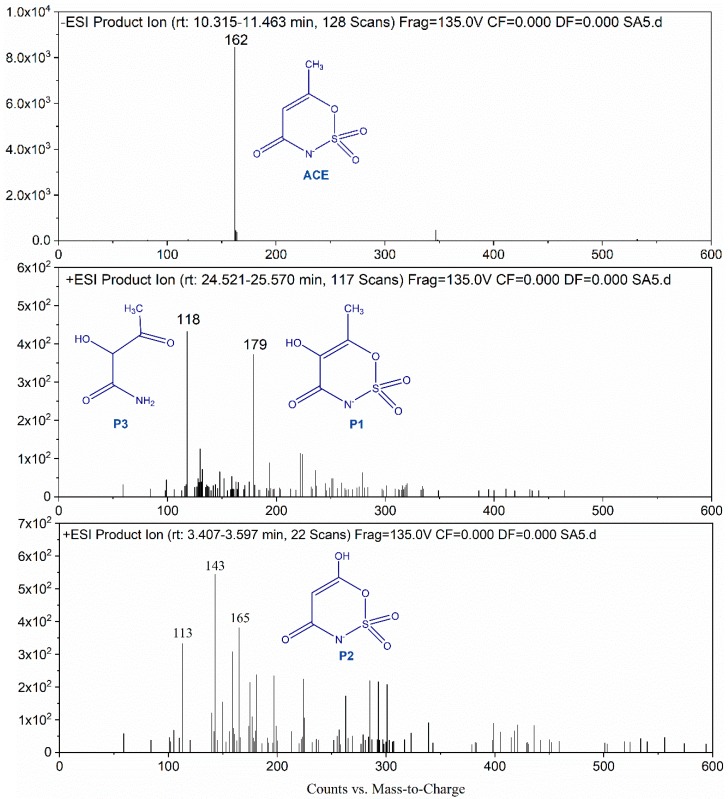
LC-MS spectra of ACE and its degradation products.

As shown in the mass spectra (Figure 6), several obvious peaks (*m/z* = 117, 164, 178) were observed, indicating that ACE was transformed into several intermediates. The ACE molecular possessed a charge-to-mass ratio (*m/z*) of 162. Like the previous studies [29], a hydroxylated product of ACE (*m/z* 179.1, P1) was detected in present work. ${\mathrm{SO}}_{4}^{•-}$ was ready to undergo reaction with organic pollutants through electron transfer. Sulfate radicals react with the olefinic double bond of ACE and form short-lived sulfate radical adducts [30]. Then nucleophilic attack of water and oxygen on the ${\mathrm{SO}}_{4}^{•-}$ adducts results in the formation of hydroxylated product. Hydroxylated product can also be generated via electron transfer from the double bond to ${\mathrm{SO}}_{4}^{•-}$, causing the formation of the intermediate radical [31]. The latter reacts with water and oxygen to produce the hydroxylated product too. The HO• attack on the organic molecular mainly follows or electrophilic addition or hydrogen abstraction mechanism. The detected hydroxylated product can be formed by HO• addition on double bond and dehydration [32]. In addition, the intermediate with an *m/z* of 165.1 (P2) was also identified in the oxidation processes. This product can be formed through HO• addition on double bond and demethylation. Besides these two products, an intermediate with *m/z* of 118.1 (P3) appeared in the mass spectra. Such intermediate can be produced from P2 decomposition through break of C-O and C-N bonds. Based on the information of these identified products, HO• and ${\mathrm{SO}}_{4}^{•-}$ are believed to involve in the degradation of ACE and the attack sites are C=C, C-O, and C-N bonds.

### 2.3. Effect Water Matrix Components on ACE Degradation

**Figure 7:**
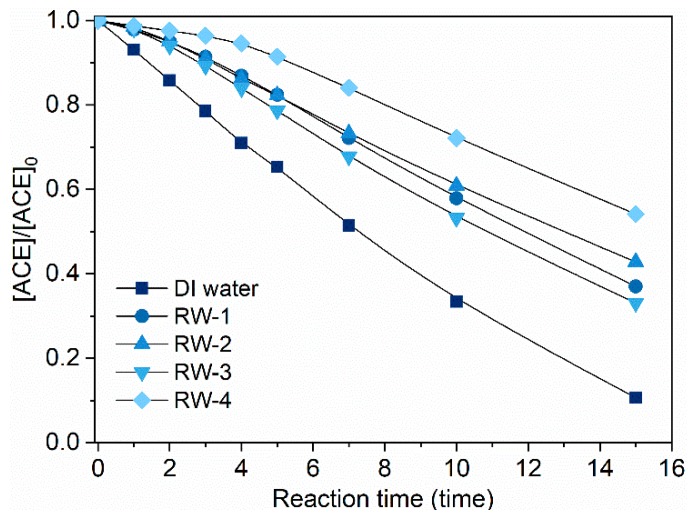
Degradation of ACE by O_3_/PMS under background of four real waters. Conditions: [ACE]_0_ = 8.0 mg·L^−1^; pH = 7.4; 15 ± 1 °C; [PMS]_0_ = 0.4 mM; O_3_ dosage 1.25 ± 0.1 µM∙min^−1^ (60 ± 5 µg∙min^−1^)).

Considering the possible scavenging effects of background water matrices, the degradation performance of ACE by O_3_/PMS in four real waters was also tested. Table 1 summarizes the water quality parameters of these four real waters (RWs). They are significantly different in indexes of dissolved organic matters (DOC), alkalinity, ${\mathrm{Cl}}^{-}$, ${\mathrm{NO}}_{3}^{-}$, ${\mathrm{SO}}_{4}^{2-}$, and ${\mathrm{Ca}}^{2+}$. As shown in Figure 7, the degradation rates of ACE in RWs generally suffered some extent of decrease compared to the case of DI water. It may result from scavenging of HO• and ${\mathrm{SO}}_{4}^{•-}$ by cosolutes like natural organic matters (NOM) and bicarbonate (${\mathrm{HCO}}_{3}^{-}$). Such significant inhibition of ACE degradation by background cosolutes makes screening of main inhibitors in real waters necessary. Thus, we evaluate the effects of possibly relevant water quality parameters one by one. 

**Figure 8:**
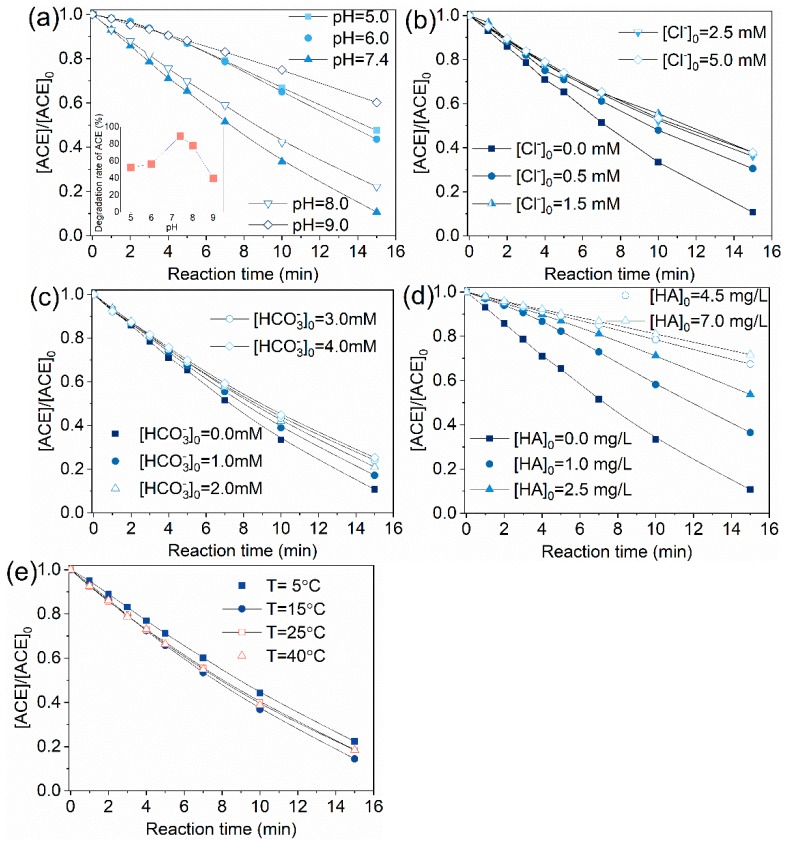
Effect of water quality parameters on the degradation of ACE using the O_3_/PMS process: (**a**) pH; (**b**) Cl^−^; (**c**) HCO_3_^−^; (**d**) HA; (**e**) temperature. Conditions: [ACE]_0_ = 8.0 mg·L^−1^; 15 ± 1 °C; [PMS]_0_ = 0.4 mM; O_3_ solution dosing rate 1.25 ± 0.1 µM∙min^−1^ (60 ± 5 µg∙min^−1^).

ACE degradation efficiency increased with pH elevation in the pH range 5.0–7.4 and the removal rate dropped from 89.3% to 77.9% as the pH further increased from 7.4 to 8.0 (Figure 8a). The increase of pH from 8.0 to 9.0 made the degradation rate decrease to 39.8%. These results indicated that the most efficient degradation of ACE by O_3_/PMS is under neutral condition (insert in Figure 8a). Notably, Yang et al. [23] found that degradation of nitrobenzene and atrazine were promoted with increasing pH, which is different from what we observed here. Based on Equations (1)–(6), the primary precursors of ${\mathrm{SO}}_{4}^{•-}$/HO• are ${\mathrm{SO}}_{5}^{•-}$/$O_{3}^{•-}$ and the increasing pH will inhibit the formation of HO•, which cannot explain the phenomena observed in present work. According to the mechanism proposed by Tomiyasu, Fukutomi, and Gordon (TFG mechanism) [33], O_3_ can react with ${\mathrm{OH}}^{-}$ to produce hydroperoxide (${\mathrm{HO}}_{2}^{-}$) under neutral or alkaline condition (Equation (13), [26]). Similarly, PMS can also react with ${\mathrm{OH}}^{-}$ to form ${\mathrm{HO}}_{2}^{-}$ (Equation (14), [34]). Hydroperoxide reacts with O_3_ and PMS to generate HO• (Equation (15), [26]) and ${\mathrm{SO}}_{4}^{•-}$ (Equation (16), [13], respectively. Because PMS was in excess over O_3_, the formed ${\mathrm{HO}}_{2}^{-}$ was believed to mainly react with PMS. In addition, conversion of ${\mathrm{SO}}_{4}^{•-}$ to HO• and HO• to $O^{•-}$ is weak under conditions of pH<9.0 according to previous work (Equation (17)–(19), [20]). In summary, when the solution pH shifted from neuter to alkaline region, the proportion of O_3_ directly reacting with ACE dropped, leading to enhanced formation of ${\mathrm{SO}}_{4}^{•-}$ and suppressed formation of HO•. Given that ${\mathrm{SO}}_{4}^{•-}$ degraded ACE more slowly than HO• did (Equations (20)–(21), [35]), the oxidation capacity of the system was weakened due to the decrease of HO•. Thus, we can reasonably explain the inhibition effect caused by pH increase from 7.4 to 9.0.

(13) $$O_{3}+{\mathrm{OH}}^{-}\to {\mathrm{HO}}_{2}^{-}+O_{2}\;70{\;M}^{-1}\cdot s^{-1}$$

(14) $${\mathrm{HSO}}_{5}^{-}+{\mathrm{OH}}^{-}\to {\mathrm{HO}}_{2}^{-}+{\mathrm{SO}}_{4}^{2-}+H^{+}$$

(15) $$O_{3}+{\mathrm{HO}}_{2}^{-}\to {\mathrm{HO}}^{•}+O_{2}^{•-}+O_{2}\;2.8\times 10^{6}{\;M}^{-1}\cdot s^{-1}$$

(16) $${\mathrm{HSO}}_{5}^{-}+{\mathrm{HO}}_{2}^{-}\to {\mathrm{SO}}_{4}^{•-}+O_{2}^{•-}+H_{2}O\;M^{-1}\cdot s^{-1}$$

(17) $${\mathrm{SO}}_{4}^{•-}+{\mathrm{OH}}^{-}\to {\mathrm{HO}}^{•}+{\mathrm{SO}}_{4}^{2-}\;6.5\times 10^{7}{\;M}^{-1}\cdot s^{-1}$$

(18) $${\mathrm{HO}}^{•}+{\mathrm{OH}}^{-}\to O^{•-}+H_{2}O\;4.0\times 10^{10}{\;M}^{-1}\cdot s^{-1}$$

(19) $$O^{•-}+H_{2}O\to {\mathrm{HO}}^{•}+{\mathrm{OH}}^{-}\;2.1\times 10^{9}{\;M}^{-1}\cdot s^{-1}$$

(20) $${\mathrm{HO}}^{•}+\mathrm{ACE}\to \mathrm{Products}\;3.8\times 10^{9}{\;M}^{-1}\cdot s^{-1}$$

(21) $${\mathrm{SO}}_{4}^{•-}+\mathrm{ACE}\to \mathrm{Products}\;<2.0\times 10^{7}{\;M}^{-1}\cdot s^{-1}$$

Presence of 0.5–5.0 mM ${\mathrm{Cl}}^{-}$ inhibited ACE degradation by O_3_/PMS process overall. It should be noted that 0.5 mM Cl^−^ showed a 19.9% inhibition but ${\mathrm{Cl}}^{-}$ at concentration of >5 mM did not cause extra inhibition effect. Under neutral pH, ${\mathrm{Cl}}^{-}$ is considered to show subtle influence on HO• concentration as the reaction forms ${\mathrm{ClOH}}^{•-}$ is reversibly and generation of Cl• occurs only at low pH conditions (Equations (22)–(24), [36]). Thus, ${\mathrm{Cl}}^{-}$ is considered to exert its influence by scavenging ${\mathrm{SO}}_{4}^{•-}$ to generate less reactive Cl• (Equations 25–26, [37]).

(22) $$\mathrm{HO}+{\mathrm{Cl}}^{-}\to {\mathrm{ClOH}}^{•-}\;4.3\times 10^{9}{\;M}^{-1}\cdot s^{-1}$$

(23) $${\mathrm{ClOH}}^{•-}\to \mathrm{HO}+{\mathrm{Cl}}^{-}\;6.1\times 10^{9}{\;M}^{-1}\cdot s^{-1}$$

(24) $${\mathrm{ClOH}}^{•-}+H^{+}\to H_{2}O+\mathrm{Cl}\;2.1\times 10^{10}{\;M}^{-1}\cdot s^{-1}$$

(25) $${\mathrm{SO}}_{4}^{•-}+{\mathrm{Cl}}^{-}\to {\mathrm{SO}}_{4}^{2-}+\mathrm{Cl}\;3.0\times 10^{8}{\;M}^{-1}\cdot s^{-1}$$

(26) $${\mathrm{SO}}_{4}^{2-}+\mathrm{Cl}\to {\mathrm{SO}}_{4}^{•-}+{\mathrm{Cl}}^{-}\;2.5\times 10^{8}{\;M}^{-1}\cdot s^{-1}$$

Similar to the case of ${\mathrm{Cl}}^{-}$, 1.0–4.0 mM ${\mathrm{HCO}}_{3}^{-}$ showed a negative effect on the degradation rate of ACE on the whole. 1.0 mM ${\mathrm{HCO}}_{3}^{-}$ showed a 10.3% inhibition and further increase of ${\mathrm{Cl}}^{-}$ concentration brought no additional inhibition effect. Equations (27)–(30) describe the reaction of HO• and ${\mathrm{SO}}_{4}^{•-}$ with ${\mathrm{HCO}}_{3}^{-}$ [38]. scavenging rates of HO• and ${\mathrm{SO}}_{4}^{•-}$ by ${\mathrm{HCO}}_{3}^{-}$ are calculated to be (8.6–34.4) × 10^3^ s^−1^ and (2.8–11.2) × 10^3^ s^−1^, while the scavenging rates of HO• and ${\mathrm{SO}}_{4}^{•-}$ by ACE are 1.52×10^3^ s^−1^ and <0.8×10^3^ s^−1^. By comparison, one can find that ${\mathrm{HCO}}_{3}^{-}$ exerted its inhibition effect through scavenging ${\mathrm{SO}}_{4}^{•-}$ and HO•. 2.0 mM ${\mathrm{HCO}}_{3}^{-}$ is enough to convert most of HO• and ${\mathrm{SO}}_{4}^{•-}$ to less active ${\mathrm{CO}}_{3}^{•-}$ and 3.0–4.0 mM ${\mathrm{HCO}}_{3}^{-}$ is overdosed. This fact may explain the observed phenomena.

(27) $$\mathrm{HO}+{\mathrm{HCO}}_{3}^{-}\to {\mathrm{CO}}_{3}^{•-}+H_{2}O\;8.6\times 10^{6}{\;M}^{-1}\cdot s^{-1}$$

(28) $$\mathrm{HO}+{\mathrm{CO}}_{3}^{2-}\to {\mathrm{OH}}^{-}+{\mathrm{CO}}_{3}^{•-}\;3.9\times 10^{8}{\;M}^{-1}\cdot s^{-1}$$

(29) $${\mathrm{SO}}_{4}^{•-}+{\mathrm{HCO}}_{3}^{-}\to {\mathrm{CO}}_{3}^{•-}+{\mathrm{HSO}}_{4}^{-}\;2.8\times 10^{6}{\;M}^{-1}\cdot s^{-1}$$

(30) $${\mathrm{SO}}_{4}^{•-}+{\mathrm{CO}}_{3}^{2-}\to {\mathrm{CO}}_{3}^{•-}+{\mathrm{SO}}_{4}^{2-}\;6.1\times 10^{6}{\;M}^{-1}\cdot s^{-1}$$

Natural organic matter (NOM) is also a common radical scavenger in real waters. Here, HA was selected as representative of NOM to research the effect of NOM. It can be seen from Figure 8d that when the HA concentration in reaction solution was 1, 2.5, 4.5, 7.0 mg·L^−1^, the degradation rate of ACE decreased from 89.3% to 63.5%, 46.3%, 32.6%, and 28.3%, respectively. HA was reported to react with HO• and ${\mathrm{SO}}_{4}^{•-}$ at rate constants of 2.5 × 10^4^ (mg·L^−1^C)^−1^·s^−1^ [39] and 9.4 × 10^3^ (mg·L^−1^C)^−1^·s^−1^ [40]. 1–7.0 mg·L^−1^ HA scavenges HO• at rates of (2.5–17.5) ×10^4^ s^−1^ and ${\mathrm{SO}}_{4}^{•-}$ at rates of (9.4–65.8) × 10^3^ s^−1^, while 8.0 mg·L^−1^ ACE captures HO• and ${\mathrm{SO}}_{4}^{•-}$ at a rate of 1.52 × 10^3^ s^−1^ and <0.8×10^3^ s^−1^, respectively. After comparing the scavenging rates of ${\mathrm{HO}}^{•}$/${\mathrm{SO}}_{4}^{•-}$ by ACE and HA, the obvious inhibition effect caused by HA is easily understood. 

As can be seen from Figure 6e, temperature was not a factor which significantly affected the ACE degradation. ACE removal rate increased slightly when temperature rose from 5 to 40 ℃. These results indicate that O_3_/PMS process is not thermodynamically controlled in the investigated temperature range. This is quite similar to O_3_/H_2_O_2_, which was almost not influenced by reaction temperature [41].

### 2.4. EE/O Analysis

In order to determine whether O_3_/PMS is cost-effective for a given situation, EE/O concept was applied [42]. The electrical energy related to O_3_ and PMS consumption ($\mathrm{EE}/O_{O_{3}}$ and $\mathrm{EE}/O_{\mathrm{PMS}}$) which is required for an order of ACE removal (i.e., 90% destruction of ACE) were calculated using Equations (31)–(33):

(31) $$\mathrm{EE}/O_{\mathrm{total}}=\mathrm{EE}/O_{O_{3}}+\mathrm{EE}/O_{\mathrm{PMS}}$$

(32) $$\mathrm{EE}/O_{O_{3}}=\left[O_{3}\right]\times k_{O_{3}}\times 1000/(V\times \log\frac{{\left[\mathrm{ACE}\right]}_{0}}{{\left[\mathrm{ACE}\right]}_{t}})$$

(33) $$\mathrm{EE}/O_{\mathrm{PMS}}=\left[\mathrm{PMS}\right]\times k_{\mathrm{PMS}}\times 1000/(V\times \log\frac{{\left[\mathrm{ACE}\right]}_{0}}{{\left[\mathrm{ACE}\right]}_{t}})$$

where [O_3_] and [PMS] are the amounts of O_3_ and PMS consumption with the unit of g·L^−1^, $k_{O_{3}}$ and $k_{\mathrm{PMS}}$ are the electrical energy consumption per kg O_3_ and PMS in kWh·kg^−1^. [ACE]_0_ is the initial concentration of ACE and [ACE]_t_ is the concentration at reaction time t with the unit of mM. V is the volume of reactor with the unit of L. $\mathrm{EE}/O_{O_{3}}$ and $\mathrm{EE}/O_{\mathrm{PMS}}$ are calculated to be 1.875 kWh·m^−3^ and 2.7 kWh·m^−3^, leading to a $\mathrm{EE}/O_{\mathrm{total}}$ value of 4.575 kWh·m^−3^ (Table 2). The EE/O value of O_3_/PMS is comparable to that of UV/PMS (6.8 kWh·m^−3^, [43]) or UV/H_2_O_2_ (7.8 kWh·m^−3^, [43]) but much higher than that of O_3_/H_2_O_2_ (<1.0 kWh·m^−3^, [44]). 

## 3. Materials and Methods 

### 3.1. Materials

Acesulfame potassium (98%) was purchased from Adamas Reagent Co., Ltd. (Shanghai, China). Potassium peroxymonosulfate (PMS) and humic acid (HA) were American Chemical Society (ACS) reagent grade and were obtained from Sigma-Aldrich (San Francisco, CA, USA). 5,5-dimethyl-1-pyrroline N-oxide (DMPO) (98%), ${\mathrm{Na}}_{2}B_{4}O_{7}\cdot 10H_{2}O$ (99.5%), and $H_{3}{\mathrm{BO}}_{3}$ (ACS reagent, 99.5%) were ordered from J&K Scientific (Beijing, China). Indigo carmine (90%) and ${\mathrm{NaNO}}_{2}$ (99%) were analytical reagent and traceable to Aladdin (Shanghai, China). HClO_4_ (70–72%) and ethanol (HPLC grade) were ordered from Sinopharm Chemical Reagent Co., Ltd. (Shanghai, China). Ammonium acetate, tert-butanol (TBA), NaOH, NaHCO_3_, NaCl were all analytical-reagent and traceable to Sinopharm Chemical Reagent Co., Ltd. (Shanghai, China). High-performance liquid chromatography (HPLC)-grade methanol was obtained from Fisher Scientific (Waltham, MA, USA). Ultrapure water (18.2 MΩ⋅cm) was used to prepare solutions. Four natural water samples were collected from different cities of Zhejiang province (China). The water samples were dechlorinated before use.

HA stock solution was prepared in a procedure similar to that described in our previous work [45]. The accurate concentration of the HA stock solution was calibrated using a total organic carbon (TOC)-VCPH analyzer (Shimadzu, Japan).

### 3.2. Experimental Procedures

A Guolin CF-G-3-10g ozone generator (Qingdao, China) was used to produce O_3_. Then O_3_ stock solution was prepared by bubbling O_3_ into 1500 ml DI water of pH = 4.0 (adjusted with HClO_4_) which was cooled by ice bath. The experiment was conducted in a 500-mL glass reactor. ACE (8.0 mg∙L^−1^) was initially prepared with ultrapure water. HClO_4_/NaOH (0.1 M) was used to adjust pH value from 5.0–6.0 and 2 mM borate buffer was used to adjust pH value from 7.4 to 9.0. A magnetic stirrer was used to mix the reaction solution evenly throughout the whole process. Using a water bath to maintain the temperature of the reaction solution at 15 °C so as to slow down the decomposition of O_3_ itself. PMS solution (100 mM) was then added to generate an initial concentration of 0.4 mM. At the same time, O_3_ solution was added to the reaction system by a peristaltic pump (Longer, Baoding, China) at a dosing rate of 60 ± 5 µg∙min^−1^. Timing was started simultaneously. The O_3_ concentration was determined immediately before and after the reaction, and the average of the two concentrations was taken to calculate the dosage of O_3_. The residual oxidant in each sample was removed by NaNO_2_ before HPLC analysis.

### 3.3. Analysis Methods

The concentration of O_3_ solution was determined by indigo method [46]. The absorbance at the wavelength of 612 nm was detected by a Hach DR6000 ultraviolet–visible spectrophotometer (Hach, Loveland, CO, USA). ACE was quantified by an Agilent 1200 HPLC (Agilent, Palo Alto, CA, USA). Separation was performed with an Agilent Eclipse XDB-C18 column (5 μm, 4.6 × 150 mm) at 30 °C. The mobile phase consisted of 90% ammonium acetate (0.02 mol∙L^−1^) and 10% methanol and had a flow rate of 1mL⋅min^−1^. Detection wavelength was set at 230 nm. 20 μL sample injection was employed. Products analysis was performed by Agilent 6460 triple-quad HPLC-MS (Agilent, Palo Alto, CA, USA). The samples were concentrated by solid phase extraction 50 times before product analysis.

Typical water quality indexes were measured for the four collected effluent samples of waterworks filter tank. Alkalinity (as ${\mathrm{CO}}_{3}^{2-}$) was quantified according to the Standard Methods for the Examination of Water and Wastewater [47]. DOC (sample were filtrated with 0.45 μm membrane) and TOC was determined via a Shimadzu TOC analyzer (Shimadzu, Kyoto, Japan). The concentrations of cations (Ca^2+^, Mn^2+^, Cu^2+^, and total Fe) were determined using a PerkinElmer NexION 350Q ICP-MS Spectrometer (PerkinElmer, Shelton, CT, USA). The ${\mathrm{Cl}}^{-}$ and ${\mathrm{NO}}_{3}^{-}$ measurements were carried out via a Dionex ICS-2000 ion chromatograph (Chameleon 6.8, Sunnyvale, CA, USA). UV absorbance at 254 nm (UV_254_) was determined with a Shimadzu UV-250 spectrophotometer (Shimadzu, Kyoto, Japan). The pH was determined using an Orion 3-Star pH meter (Thermo Fisher, Shanghai, China). 

A Bruker A200 electron paramagnetic resonance (EPR) 300E instrument (Bruker, Karlsruhe, Germany) was used to qualitatively analyze HO• and ${\mathrm{SO}}_{4}^{•-}$. 5,5-dimethyl-1-pyrroline-N-oxide (DMPO) was used as a spin-trapping agent. The desired concentrations of DMPO, O_3_, and PMS were mixed for 1 min and transferred into a 200 mL capillary tube for EPR test. The EPR spectrometer settings in the spin trapping experiments were as follows: modulation amplitude, 0.1 mT; center field, 351.194 mT; sweep width, 10.00 mT; sweep time, 41 s; microwave power, 2.25 mW; microwave frequency, 9.858 GHz; and receiver gain, 1.42 × 10^4^.

## 4. Conclusions

In this study, the ACE degradation by the system of O_3_/PMS was studied in detail. It was demonstrated that efficient degradation of ACE was achieved due to the coaction of O_3_, HO•, and ${\mathrm{SO}}_{4}^{•-}$. The degradation progress was significantly affected by several factors including the dosing ratio of PMS and O_3_, the pH value, ${\mathrm{Cl}}^{-}$ concentration, and NOM concentration. The obtained optimum operational conditions included a reaction pH 7.4 and 0.4 mM PMS: 60 ± 5 µg O_3_∙min^−1^. Identified intermediates evidenced that the attack sits of ACE by oxidative species are C==C, C-O, and C-N bonds. EE/O analysis of ACE degradation by O_3_/PMS demonstrated that the PMS consumption accounted for the largest proportion of total cost.

## Figures and Tables

**Table 1:** Water quality of the four real waters (RWs).

Water Matrices	Units	RW-1	RW-2	RW-3	RW-4
pH		7.3	7.5	7.2	7.5
DOC	mg C·L^−1^	2.80	4.41	1.93	4.45
Alkalinity (as CO_3_^2−^)	mg·L^−1^	24	10.31	7.89	7.26
Cl^−^	mg·L^−1^	4.21	57.2	15.708	3.653
NO_3_^−^	mg·L^−1^	0.802	1.480	9.392	6.573
UV_254_	cm^−1^·(mg·L^−1^)^−1^	0.008	0.020	0.015	0.108
SO_4_^2−^	mg·L^−1^	6.970	55.0	18.331	26.481
Ca^2+^	mg·L^−1^	44.5	137	8.253	--
Mn^2+^	mg·L^−1^	2.48 × 10^−3^	<0.05	0.012	--
Cu^2+^	mg·L^−1^	7.64 × 10^−4^	<0.1	0.076	--
Total Fe	mg·L^−1^	7.21 × 10^−3^	<0.05	0.155	--

**Table 2:** Cost of O_3_/PMS for ACE degradation.

P	t	V	$EE/O_{O_{3}}$	U/P_(PMS)_	U/P_(Ele)_	C	M	$EE/O_{PMS}$	$EE/O_{total}$
(kW)	(h)	(L)	(kWh·m^−3^)	($/g)	($/kWh)	(mM)	(g·mol^−1^)	(kWh·m^−3^)	(kWh·m^−3^)
0.0036	0.25	0.48	1.875	0.0042	0.1132	0.4	307.35	2.7	4.575
